# Rapid and sensitive point-of-care detection of *Leptospira* by RPA-CRISPR/Cas12a targeting *lipL32*

**DOI:** 10.1371/journal.pntd.0010112

**Published:** 2022-01-06

**Authors:** Sirawit Jirawannaporn, Umaporn Limothai, Sasipha Tachaboon, Janejira Dinhuzen, Patcharakorn Kiatamornrak, Watchadaporn Chaisuriyong, Jom Bhumitrakul, Oraphan Mayuramart, Sunchai Payungporn, Nattachai Srisawat

**Affiliations:** 1 Excellence Center for Critical Care Nephrology, King Chulalongkorn Memorial Hospital, Bangkok, Thailand; 2 Ph.D. candidate in Doctor of Philosophy Program in Medical Sciences, Faculty of Medicine, Chulalongkorn University, Bangkok, Thailand; 3 Critical Care Nephrology Research Unit, Faculty of Medicine, Chulalongkorn University, Bangkok, Thailand; 4 King’s College London GKT School of Medical Education, King’s College London, London, England, United Kingdom; 5 Research Unit of Systems Microbiology, Department of Biochemistry, Faculty of Medicine, Chulalongkorn University, Bangkok, Thailand; 6 Division of Nephrology, Department of Medicine, Faculty of Medicine, Chulalongkorn University, and King Chulalongkorn, Memorial Hospital, Bangkok, Thailand; 7 Academy of Science, Royal Society of Thailand, Bangkok, Thailand; 8 Tropical Medicine Cluster, Chulalongkorn University, Bangkok, Thailand; 9 Excellence Center for Critical Care Medicine, King Chulalongkorn Memorial Hospital, Bangkok, Thailand; 10 Department of Critical Care Medicine, Center for Critical Care Nephrology, The CRISMA Center, University of Pittsburgh School of Medicine, Pennsylvania, United States of America; UConn Health, UNITED STATES

## Abstract

**Background:**

One of the key barriers preventing rapid diagnosis of leptospirosis is the lack of available sensitive point-of-care testing. This study aimed to develop and validate a clustered regularly-interspaced short palindromic repeat (CRISPR)/CRISPR-associated protein 12a (CRISPR/Cas12a) platform combined with isothermal amplification to detect leptospires from extracted patient DNA samples.

**Methodology/Principal findings:**

A Recombinase Polymerase Amplification (RPA)-CRISPR/Cas12a-fluorescence assay was designed to detect the *lipL32* gene of pathogenic *Leptospira* spp. The assays demonstrated a limit of detection (LOD) of 100 cells/mL, with no cross-reactivity against several other acute febrile illnesses. The clinical performance of the assay was validated with DNA extracted from 110 clinical specimens and then compared to results from qPCR detection of *Leptospira* spp. The RPA-CRISPR/Cas12a assay showed 85.2% sensitivity, 100% specificity, and 92.7% accuracy. The sensitivity increased on days 4–6 after the fever onset and decreased after day 7. The specificity was consistent for several days after the onset of fever. The overall performance of the RPA-CRISPR/Cas12a platform was better than the commercial rapid diagnostic test (RDT). We also developed a lateral flow detection assay (LFDA) combined with RPA-CRISPR/Cas12a to make the test more accessible and easier to interpret. The combined LFDA showed a similar LOD of 100 cells/mL and could correctly distinguish between known positive and negative clinical samples in a pilot study.

**Conclusions/Significance:**

The RPA-CRISPR/Cas12 targeting the *lipL32* gene demonstrated acceptable sensitivity and excellent specificity for detection of leptospires. This assay might be an appropriate test for acute leptospirosis screening in limited-resource settings.

## Author summary

Clinical signs and symptoms of leptospirosis are similar to those of other infectious diseases such as dengue, sepsis, and malaria, making it difficult to diagnose. In this study, we developed an RPA-CRISPR/Cas12a -based detection platform to identify the *lipL32* gene of pathogenic *Leptospira* spp. The results showed that the limit of detection (LOD) was approximately 10^2^ cells/mL without cross-reactivity against other infectious diseases. The platform was validated using 110 patients from 15 hospitals in Sisaket province, Thailand. The sensitivity, specificity, and accuracy was found to be 85.2%, 100% and 92.7%, respectively, for the diagnosis of leptospirosis. Assay sensitivity increased at 4–6 d post-onset of fever, with a consistent specificity every day after the onset of fever. We also developed a lateral flow detection assay combined with RPA-CRISPR/Cas12a, which also had a LOD of 10^2^ cells/mL and could correctly distinguish known positive and negative clinical samples in a pilot study. Findings from this study demonstrate the potential effectiveness of the RPA-CRISPR/Cas12a platform in improving speed and accuracy of leptospirosis diagnosis especially in limited-resource settings.

## Introduction

Leptospirosis is a zoonotic disease that affects global health with over one million cases and 58,900 deaths annually [1]. The disease is caused by the pathogenic spirochete *Leptospira* spp. which can adapt to a broad spectrum of mammalian hosts and environments [2,3]. The clinical signs and symptoms of leptospirosis share similarities with other infectious diseases such as dengue, sepsis, and malaria, making it difficult to diagnose [4–6].

One of the key barriers in reducing the impact of leptospirosis is the lack of affordable sensitive diagnostic tools. There are currently standard methods recommended by the WHO [4]. The first is the microscopic agglutination test (MAT), a serological-based diagnosis method. Although the MAT is accurate, it requires a skilled technician, well-equipped laboratory, and is time-consuming. The second is dark field microscope diagnosis from sample cultures collected from the patient’s blood at the early stage of *Leptospira* spp. infection. However, because *Leptospira* spp. is a slow-growing bacterium, results can take several weeks. The third technique is the use of polymerase chain reaction (PCR), a nucleic acid detection method that is faster and more accurate. The quantitative polymerase chain reaction (qPCR) is an improvement of the PCR that is widely used as the primary diagnostic procedure [5]. The drawback of qPCR is the high-cost of equipment and the lack of availability in every hospital, especially in rural areas [5,6] where most leptospirosis cases occur. The WHO also recommends the enzyme-linked immunosorbent assay (ELISA) for antibody detection. However, antibody levels are often low during the first week after infection [5]. The development of a novel diagnostic tool that can detect pathogenic *Leptospira* spp. early, providing rapid and accurate results and is not cost-prohibitive to resource-limited hospitals and clinics is essential for public health [7].

Testing at the point of care is ideal for resource-constrained settings. Numerous studies have been conducted to investigate point-of-care tests for leptospirosis [8–10]. Loop-Mediated Isothermal Amplification for Diagnosis of both pathogenic *Leptospira* and intermediate *Leptospira* in humans by targeting *rrs* gene has been developed. However, this technique is limited due to an imprecise specificity and does not support routine clinical usage of LAMP [10]. Anti-*leptospira* IgM rapid diagnostic tests (RDTs) are commercially available. RDTs have a limited sensitivity and may be inefficient for screening acute leptospirosis [9].

Recombinase polymerase amplification (RPA) is an isothermal nucleic acid amplification technology that can be operated in the field due to its low resource requirements. The RPA system utilizes three enzymes: recombinase, single-stranded DNA-binding protein (SSB), and strand-displacing polymerase [11]. The recombinase can pair oligonucleotide primers with homologous sequences in the target DNA. The SSB then binds to the replaced strand of DNA and protects the dissociation of primers. The strand-displacing polymerase initiates DNA synthesis. Amplification of the target DNA sequence by RPA can be accomplished at a constant temperature in less than 20 min. Moreover, RPA can work with the clustered regularly interspaced short palindromic repeats (CRISPR)/Cas12a system that has shown promising results in nucleic acid detection [12–14]. The CRISPR/Cas12a system relies on a crispr RNA (crRNA) which acts as a targeting system for the effector function of the Cas12a enzyme to recognize and cleave specific DNA targets. After CRISPR/Cas12a detects its target and cleaves it, the collateral cleavage activity is activated resulting in the fluorescent reporter being cleaved from the quencher and creating a detectable fluorescent signal [14]. For this reason, RPA preamplification combined with the CRISPR/Cas12a detection system can be used for diagnostic screening in a limited-resource setting without the need for specialized instruments. The purpose of this study was to develop a new early rapid leptospirosis diagnostic tool using RPA and CRISPR/Cas12a targeting *lipL32*, which is considered to be a pathogenic species-specific gene that plays a critical role in inflammatory responses via binding to Toll-like receptor 2 [15,16]. Furthermore, the *lipL32* gene is highly conserved across pathogenic *Leptospira* species [17].

## Materials and methods

### Ethics statement

The study protocol was approved by the Institutional Review Board of the Faculty of Medicine, Chulalongkorn University, Bangkok, Thailand (IRB No.655/63). The study was performed under the international guidelines for human research protection of the Declaration of Helsinki, The Belmont Report, CIOMS Guideline, and International Conference on Harmonization in Good Clinical Practice. Written informed consent was obtained from each participant to participate in this study.

### Culturing *Leptospira interrogans*

For the direct culture of *Leptospira interrogans* that was used as a positive control and limit of detection for the study, 1 mL of fresh whole blood was added into 4 mL of Ellinghausen, McCullough, Johnson, and Harris (EMJH) medium and incubated at 30°C for two weeks. The culture was examined using dark field microscopy to confirm the existence of *Leptospira* (18, 19). After confirmation, *Leptospira* cells were counted in a Petroff-Hauser chamber as previously described in [18,19]. DNA were then extracted at 10^8^ cells/mL using the High Pure PCR Template Preparation Kit (Roche, USA).

### Patients and study design

In this study, we tested the performance of the RPA-CRISPR/Cas12a system targeting *lipL32* gene using blood samples from participants with a known leptospirosis status (infected or non-infected) from previous studies conducted in 15 hospitals in Sisaket province, Thailand (THAI-LEPTO study) [20]. The samples were collected between December 2015 to November 2016. The sample size was calculated when the sensitivity or specificity value of the standard method (qPCR) was known which is 86% and 100%, respectively [21]. A minimum of 110 samples were determined to be needed for this study according to the calculations.

Inclusion criteria for subjects were as follows: (i) older than 18 years old and admitted to a participating hospital; (ii) presenting with clinical suspicion of leptospirosis, high fever (body temperature higher than 38°C), severe myalgia; and (iii) history of exposure to reservoir animals [9,22]. The exclusion criteria were patients who suffered from other known infectious diseases. Following blood collection, total DNA was extracted from 200μL of whole blood samples for clinical sample validation using the High Pure PCR Template Preparation Kit (Roche, USA) according to the manufacturer’s instructions. A NanoDrop 2000 was used to determine the concentration and quality of the extracted DNA (Thermo Scientific, USA). Separation of serum from whole blood was accomplished by centrifuging at 3000 rpm for 10 minutes and storing at -80^o^ C until the rapid diagnostic test (RDT) was performed. The samples from the first day of enrollment were selected and used as a blind test.

### Detection by qPCR assay

Each positive sample based on the qPCR assay was defined as a leptospirosis confirmed case. The qPCR targeting the *lipL32* gene was performed as previously described with minor modification (24). Briefly, 242 base pair products were amplified and detected using the forward primer (5′- AAG CAT TAC CGC TTG TGG TG -3′), reverse primer (5′- GAA CTC CCA TTT CAG CGA TT -3′) and *Taq*man probe (5′-FAM- AA AGC CAG GAC AAG CGC CG-BHQ1-3′). The qPCR mixture consisted of 5 μL of extracted DNA, 10 μL of SsoAdvanced Universal Probe Supermix (Bio-Rad Laboratories, USA), 1 μL of each primer (10 μM), 0.4 μL of *Taq*man probe (10 μM), and 2.6 μL of nuclease-free water in a final volume of 20 μL. The qPCR reactions were performed in duplicate. A no template control (NTC) with all the above reagents was used as the negative control. Extracted DNA of *Leptospira interrogans* from EMJH culture was used as positive control. Amplification and fluorescence detection were conducted in the StepOnePlus Real-Time PCR System (Applied Biosystems, USA). The amplification protocol consisted of 10 min at 95°C, followed by 45 cycles of 15 s at 95°C and 1 min at 60°C. A negative result was considered with the threshold cycle (Ct) value higher than 40 cycles.

### The RPA

The *lipL32* gene amplification was performed using the TwistAmp Basic Kit (TwistDx, United Kingdom) using the same primer set as the qPCR. In brief, lyophilized RPA was resuspended in rehydration buffer and mixed with 480 nM of each primer. Then, 14 mM of magnesium acetate (final concentration) and 1 μL of extracted DNA were added to the reaction mixture. The *lipL32* gene was amplified by incubating at 39°C for 40 min, followed by heat inactivation at 75°C for 5 min.

### CRISPR RNA preparation

We designed the CRISPR RNA (crRNA) using in-silico analysis and the basic local alignment search tool (BLAST) to specifically detect the *lipL32* gene sequence adjacent to the TTTN protospacer adjacent motif (PAM) site. The *lipL32* crRNA sequence was 5′-UAAUUUCUACUAAGUGUAGAUUUCUGAGCGAGGACACAAUC-3’, consisting of a scaffold 5′-UAAUUUCUACUAAGUGUAGAU-3’ and a guide RNA 5′-UUCUGAGCGAGGACACAAUC-3’, both of which were synthesized using the HiScribe T7 High Yield RNA Synthesis Kit (New England Biolabs, UK). For the preparation of crRNA, synthetic oligonucleotides were ordered as ultramer DNA (Macrogen, South Korea) with an appended T7 promoter sequence. Oligonucleotides for crRNA (1 μM) were annealed to a short T7 primer (final concentration of 10 μM each) and incubated with T7 polymerase at 37°C for 2 h. The crRNA was then purified using a Monarch RNA Cleanup Kit (New England Biolabs, UK). The concentration of purified crRNA product was measured using a Qubit miRNA assay kit and Qubit 4 Fluorometer (Thermo Scientific, USA) and stored at -80°C until further use.

### CRISPR/Cas12a-fluorescent based detection assay (FBDA)

The CRISPR/Cas12a-FBDA performed as described previously with minor modifications.[23,24] The CRISPR/Cas12a reaction was composed of 30 nM of crRNA, 330 nM of EnGen *Lba* Cas12a (Cpf1) (New England Biolabs, USA), 600 nM of fluorescent probe (5′-FAM-TTATTATT-BHQ1-3′), 1X of NEBuffer 2.0 (New England Biolabs, USA), and 1 μL of RPA amplicons in a total reaction volume of 15 μL. The CRISPR/Cas12a reaction was incubated at 39°C for 20 min. The fluorescent signal was then observed by naked eye using a BluePAD Dual LED Blue/White Light Transilluminator (BIO-HELIX, Taiwan) at 470 nm wavelength. Each test was observed by three certified laboratory technicians who were instructed to identify the qualitative test outcome as positive or negative. The tests were considered positive if at least two of the three technicians read the results as positive. Both investigators and the technicians were unaware of the outcome.

### Limit of detection (LOD) and cross-reactivity testing

The analytic sensitivity of the assay was determined using genomic DNA isolated from *Leptospira interrogans* in EMJH cultures. Serial dilutions of genomic DNA were made from 10^6^ cells/mL down to 1 cell/mL [25]. The LOD was determined by detecting the fluorescent signal in the tube containing the lowest cells. The specimens obtained from patients with an acute febrile illness, including acute viral hepatitis, cellulitis, scrub typhus, systemic bacterial infection, acute cystitis, influenza, *Escherichia coli* septicemia, and dengue hemorrhagic fever, were tested to establish the analytical specificity of the RPA-CRISPR/Cas12a-FBDA.

### RPA-CRISPR/Cas12a combined with a lateral flow detection assay (LFDA) pilot study

A lateral flow test strip was developed to improve the RPA-CRISPR/Cas12a test and make it easier to use and read. The FITC-biotin reporter molecule and lateral flow strips were designed to capture labeled nucleic acids. The DNA probe (5′- FITC-AGGACCCGTATTCCCA-BIOTIN -3′) was used at 12 nM instead of the fluorescence probe at 600 nM under the otherwise same condition as the FBDA above. The reaction was incubated at 39°C for 30 min. The reaction was then mixed with 100 μL of running buffer and pipetted into the commercial lateral flow strip test (Kestrelbioscience, Thailand). Uncleaved reporter molecules were captured at the first detection line (test line), whereas the indiscriminate ssDNA cleavage activity of CRISPR/Cas12a did not generate a signal at the first detection line, but only a signal at the second line (control line).

### Rapid diagnostic testing

The analytic sensitivity of the RPA-CRISPR/Cas12a detection system was compared with a commercial rapid diagnostic test (RDT). A total of 96 blood samples were tested with the RDT from the Medical Science Public Health (Department of Medical Sciences, Ministry of Public Health, Thailand). The RDT kit was designed to detect anti-*Leptospira* IgM antibodies and was used according to the manufacturer’s instructions. First, the blood sample was thawed at room temperature and added to the sample well without air bubbles. Next, the assay diluent was added to the diluent well. The results were read at the end of 15 min by three trained technicians. The tests were considered positive if at least two of three technicians read the results as positive.

### Statistical analysis

Continuous variables are shown as the mean ± one standard deviation (SD) in case of a normal distribution and as a median and interquartile range (IQR) in case of non-normally distributed variables. The Student’s t-test or Mann-Whitney test was used to analyze the differences between two continuous variables. Categorical variables were presented as numbers with percentages and were compared using the Chi-square test. The performance of the RPA-CRISPR/Cas12a targeting the *lipL32* gene detection system was expressed by calculating the sensitivity, specificity, accuracy, and positive and negative predictive values compared to the qPCR analysis of the same samples. All statistical analyses were performed using the SPSS Version 22 software (SPSS, Chicago, IL).

### Analysis of the crRNA target sequence using bioinformatics

The *lipL32* sequences of *Leptospira* spp. were acquired from the National Center for Biotechnology Information (NCBI) to identify the extent of homology in the crRNA target sequences of *Leptospira* spp.

## Results

### The LOD and cross-reactivity of the RPA-CRISPR/Cas12a-FBDA

**Fig 1:**
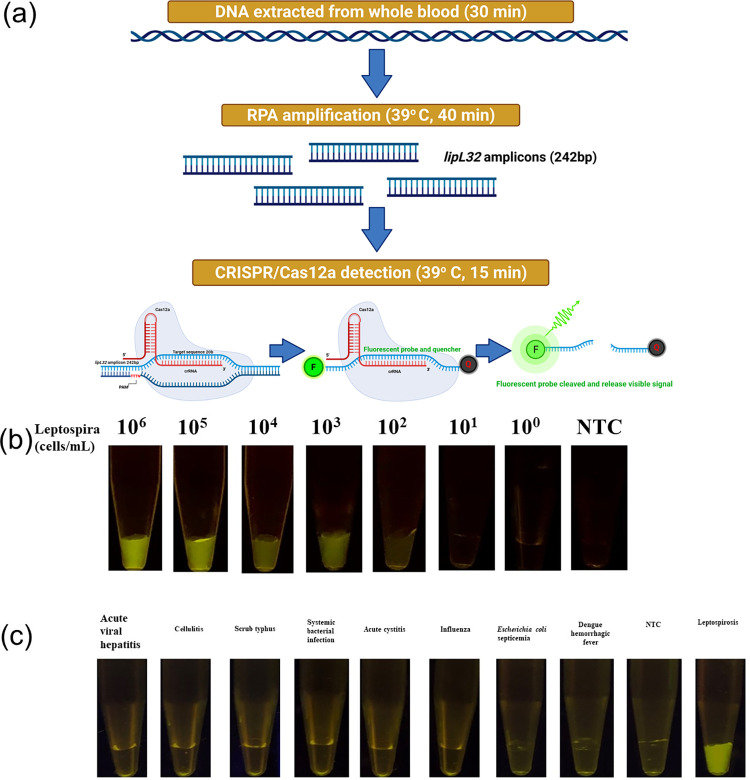
Detection of leptospirosis using the RPA-CRISPR/Cas12a-FBDA. (A) Schematic representation of the RPA-CRISPR/Cas12-FBDA’s workflow (Created with BioRender.com). (B, C) The (B) LOD and (C) cross-reactivity of the RPA-CRISPR/Cas12a targeting *lipL*32 gene against several infectious diseases with similar clinical manifestations as leptospirosis.

The LOD of the RPA-CRISPR/Cas12a-FBDA was tested by extracting DNA from a *Leptospira interrogans* culture that was serially diluted from 10^6^ to 1 cell/mL. The diluted DNA was amplified using RPA, followed by the CRISPR/Cas12a-FBDA. The workflow of the assay is summarized in Fig 1A. The LOD was found to be 100 cells/mL (Fig 1B). In addition, eight specimens from patients with other acute febrile illnesses, including acute viral hepatitis, cellulitis, scrub typhus, systemic bacterial infection, acute cystitis, influenza, *Escherichia coli* septicemia, and dengue hemorrhagic fever, were tested to explore potential cross-reactivity. The results showed no cross-reactivity (Fig 1C).

### Study population

The performance of the RPA-CRISPR/Cas12a-FBDA was validated with 110 blood samples from clinically suspected leptospirosis patients. Fifty-four samples (49.1%) were leptospirosis confirmed cases (positive by qPCR) and 56 (50.9%) were non-leptospirosis confirmed cases (negative by qPCR). The clinical characteristics of the enrolled patients are shown in Table 1. Compared with non-leptospirosis, leptospirosis patients had significantly higher serum levels of white blood cells, creatinine, total bilirubin, direct bilirubin, and potassium, but a lower systolic blood pressure (p<05). In addition, there was a significant difference in terms of days of fever until enrollment between the groups (p = .01). Other relevant laboratory investigations were not found to be significantly different between the two groups.

**Table 1:** Characteristics of the qPCR positive and negative groups with patient clinical and laboratory data.

Characteristic	Leptospirosis (N = 54)	Non-leptospirosis (N = 56)	Total (N = 110)	*P*-value
Male gender, n (%)	44 (81.48%)	45 (80.36%)	89 (80.91%)	0.88
Age, years, Mean (SD)	50.78 (16.71)	51.75 (15.93)	51.25 (16.26)	0.82
Days of fever until enrollment, Median (IQR)	3 (3, 5)	3 (2, 4)	3 (2, 4)	*0.01
Exposure to flood waters, n (%)	47 (85.45%)	40 (81.63%)	87 (79.09%)	0.18
Exposure to animals, n (%)	8 (14.55%)	9 (18.37%)	17 (15.45%)	0.73
Body temperature, Mean (SD)	38.14 (1.20)	38.16 (1.24)	38.15 (1.21)	0.97
SBP, mm HG, Median (IQR)	109.00 (96.00, 121.50)	120.00 (101.00, 129.50)	111.00 (100.00, 126.00)	*0.02
DBP, mm Hg, Median (IQR)	61.00 (58.00, 73.25)	68.00 (60.00, 76.75)	64.00 (60.00, 74.00)	0.10
Platelet (x 10^3^/μL), Median (IQR)	94500.00 (59500.00, 213250.00)	132000.00 (68500, 194750.00)	118500.00 (63000.00, 204000.00)	0.70
*WBC (x 10^3^/μL), Median (IQR)	10950.00 (8525.00, 14025.00)	8600.00 (5375.00, 12250.00)	10500.00 (6350.00, 13375.00)	*0.02
Creatinine, mg/dL, Median (IQR)	1.33 (1.00, 2.90)	1.10 (0.86, 1.27)	1.12 (0.94, 1.96)	*0.01
*TB, g/dL, Median (IQR)	1.40 (0.82, 3.30)	0.90 (0.50, 2.35)	1.18 (0.70, 2.90)	*0.02
*DB, g/dL, Median (IQR)	0.90 (0.46, 1.97)	0.50 (0.24, 1.55)	0.70 (0.30, 1.80)	*0.03
*SGOT, U/L, Median (IQR)	63.00 (41.50, 147.00)	64.50 (39.50, 170.00)	63.00 (41.00, 164.00)	0.91
*SGPT, U/L, Median (IQR)	59.00 (31.50, 103.50)	60.00 (33.00, 84.75)	60.00 (32.50, 96.00)	0.77
Na, mEq/L, Median (IQR)	135.00 (132.00, 138.00)	135.00 (131.45, 139.00)	135.00 (131.70, 139.00)	0.71
K, mEq/L, Median (IQR)	3.77 (3.40, 4.26)	3.50 (3.09, 3.90)	3.63 (3.26, 4.01)	*0.01
HCO_3_, mEq/L, Median (IQR)	24.00 (20.15, 25.75)	25.00 (22.25, 26.45)	24.55 (21.73, 26.00)	0.11

Abbreviations: WBC: white blood cell; TB: total bilirubin; DB: direct bilirubin; SGOT: serum glutamic oxaloacetic transaminase; SGPT: serum glutamic; pyruvic transaminase, Na: sodium; K: potassium; HCO: bicarbonate; DBP: diastolic blood pressure; SBP: systolic blood pressure. Continuous data are expressed as the mean, standard deviation (SD) or median shown with interquartile range (IQR). Categorical variables are expressed as numbers (%)

* represents *P* < 0.05.

### Diagnostic performance of the RPA-CRISPR/Cas12a-FBDA

To evaluate the diagnostic performance of the RPA-CRISPR/Cas12a-FBDA, 110 DNA samples from leptospirosis and non-leptospirosis confirmed cases were tested and results were compared to the qPCR results. The RPA-CRISPR/Cas12 assay yielded 100% specificity, 85.2% sensitivity, and 92.7% accuracy, with a positive predictive value (PPV) and a negative predictive value (NPV) of 100% and 87.50%, respectively (Table 2).

**Table 2:** Performance of the RPA-CRISPR/Cas12a-FBDA relative to qPCR detection.

Parameter	Performance of RPA-CRISPR/Cas12a
Total samples	110
True positive (TP)	46
True negative (TN)	56
False positive (FP)	0
False negative (FN)	8
Sensitivity	85.19%
Specificity	100%
Positive predictive value (PPV)	100%
Negative predictive value (NPV)	87.50%
Accuracy	92.73%

### Diagnosis accuracy at different days after the fever onset

**Fig 2:**
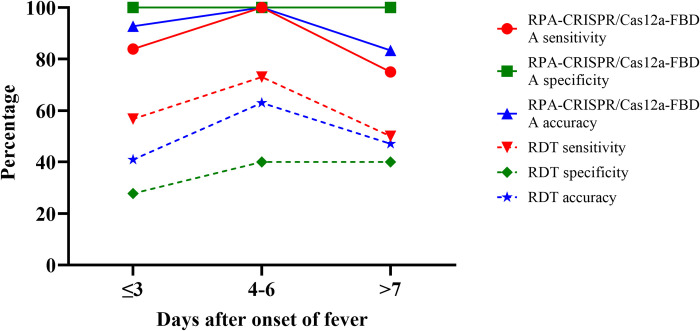
Sensitivity, specificity, and accuracy of the RPA-CRISPR/Cas12a-FBDA (solid line) and RDT (dashed line) at 3 d (red), 4–6 d (green), and ≥ 7 d (blue) after the onset of fever.

To evaluate the change in sensitivity and specificity of the assay with time after fever onset, the patients were categorized into three groups based on the time since the onset of fever (at the first day of enrollment):1) within 3 d after fever onset (n = 69), 2) within 4–6 d from fever onset (n = 19), and 3) 7 d or longer after the onset of fever (n = 18). We found that the sensitivity and accuracy of RPA-CRISPR/Cas12a-FBDA targeting *lipL32* increased on days 4–6 and decreased after day 7. In contrast, the specificity was consistent for every day after the onset of fever (Fig 2).

We also compared the diagnostic accuracy of our assay with a commercial RDT based on detection of anti-*Leptospira* IgM antibodies. We found that the commercial RDT assay yielded a lower sensitivity, specificity, and accuracy than the RPA-CRISPR/Cas12a-FBDA every day after the onset of fever (Fig 2).

### Inter-observer variability

The sensitivity, specificity, and accuracy were calculated separately to investigate each observer’s variability, with the results summarized in S1 Table. The data revealed no significant difference between observers in the ability to identify the fluorescent signal.

### The RPA-CRISPR/Cas12a LFDA

**Fig 3:**
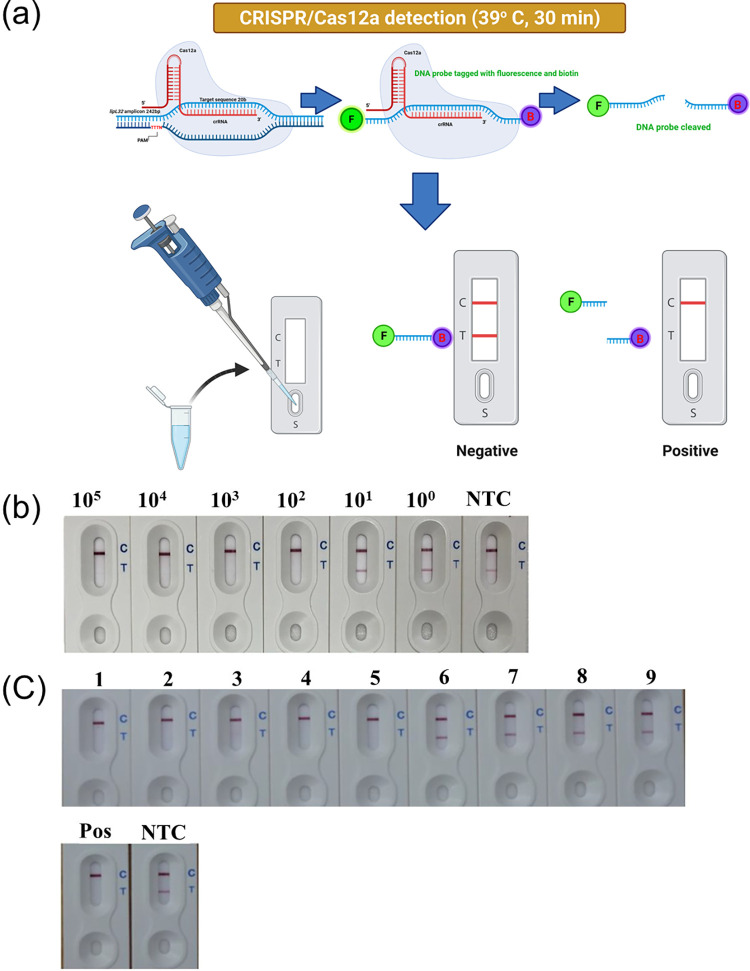
The RPA-CRISPR/Cas12a-LFDA. (A) The RPA-CRISPR/Cas12a-LFDA’s workflow (Created with BioRender.com), (B) LOD, and (C) clinical sample validation [1–5 and 6–9 are known positive and negative samples, respectively, while NTC and Pos are the no-template negative and positive control, respectively].

We also developed an RPA-CRISPR/Cas12a-LFDA (Fig 3A) to improve this test and make it more accessible for general use and easier to read. The LOD of this LFDA was 100 cells/ml similar to the FBDA (Fig 3B). Nine DNA samples from leptospirosis confirmed cases (N = 5) at a Ct between 27–37 and non-leptospirosis cases (N = 4) were tested in the pilot study. The results showed that the LFDA could reliably distinguish between the known positive and negative clinical samples (Fig 3C).

### Bioinformatics analysis of the crRNA target sequence

**Fig 4:**
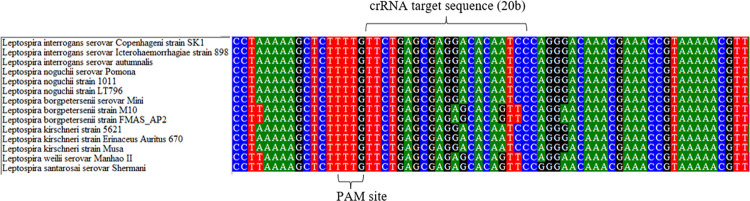
The Bioinformatics analysis of *lipL32* multiple sequence alignment between pathogenic *Leptospira*. Sequence accession numbers are as follows, CP048830.1, CP043891.1, EU526391.1, AY609326.1, AY461918.1, AY461920.1, AY609333.1, AY568680.1, NZ_CP072630.1, AY461917.1, AY461912.1, AY461911.1, AY609331.1, and CP006694.1.

The *lipL32* sequences of *Leptospira* spp. were obtained from the National Center for Biotechnology Information (NCBI) in order to determine the similarity between *Leptospira* spp. crRNA target sequences. Fig 4 and S2 Table illustrate that the majority of the sequences were found identical except for *L*. *borgpetersenii* strain M10 and FMAS_AP2, *L*. *santarosai* serovar Shermani and *L*. *weilii* serovar Manhao II.

## Discussion

The RPA-CRISPR/Cas12a-FBDA is a new nucleic acid detection platform able to diagnose many infectious diseases [12,23,24,26–29]. This study is the first report for *Leptospira* detection using the RPA-CRISPR/Cas12aFBDA assay targeting the *lipL32* gene. The assay demonstrated acceptable sensitivity (85%) and excellent specificity (100%) of *Leptospira* detection compared to qPCR as the reference test. The RPA is an isothermal nucleic acid amplification platform, which is less time-consuming than conventional PCR and qPCR [11,30]. Due to the absence of *lipL32* in nonpathogenic or intermediate *Leptospir*a spp., this newly developed test identified exclusively pathogenic *Leptospira* [16,17,31] and without cross-reactivity when tested against blood from non-leptospirosis patients.

A previous study revealed that PCR inhibitors in clinical samples can affect qPCR performance in detecting *lipL32* [32]. The RPA has the advantage of being more tolerant of PCR inhibitors making it an ideal amplification platform for this study [33]. The qPCR system provides a highly specific and sensitive tool for detecting and quantifying *Leptospira* [34]. However, due to its high cost compared to other diagnostic methods and its requirements for specialized instruments, it has not been widely used as an early diagnostic tool at the point of care.

Results from our study support usage of the RPA-CRISPR/Cas12a-based system to detect *Leptospira* with an acceptable sensitivity and high specificity. To address the sensitivity and specificity issues raised by prior studies, point-of-care testing for leptospirosis such as LAMP and RDT were created [9,10]. A previous study reported that having more than 1,000 *Leptospira* cells/mL was associated with severe leptospirosis [35]. It is notable that our assay was sensitive enough to detect *Leptospira* in the patient’s blood and administer treatment before disease symptoms became severe.

The specificity of the RPA-CRISPR/Cas12a-FBDA was found to be consistent for all three testing periods of time after the onset of fever. We reported that the sensitivity increased to 100% on days 4–6 after fever onset and decreased after day 7, which may have reflected the fact that the serum *Leptospira* spp. peaked at days 4–6 after fever onset and decreased after day 7 (39). We compared the assay performance with a commercial RDT designed for detection of anti-*Leptospira* spp. IgM antibodies. The RDT performance was similar to that previously reported [9], but had a lower sensitivity, specificity, and accuracy than the RPA-CRISPR/Cas12a-FBDA developed in this study. The window of positivity for the RPA-CRISPR/Cas12a platform began at the first sign of infection, compared to the RDT window which started at days 6–8 [5,36]. While *Leptospira* can be found in blood within the first week following the beginning of symptoms, the bacterial load diminishes over time, limiting the test’s sensitivity, whereas the IgM level begins to climb to detectable levels after the first week [36]. However, both our newly developed test and qPCR were able to detect *Leptospira* spp. after day 7 since *Leptospira* spp. can still be detected in blood albeit with a decreased number [36]. Therefore, using this RPA-CRISPR/Cas12a-FBDA targeting *lipL32* combined with RDTs would expand the window of positivity and enhance the accuracy of the leptospirosis diagnosis. We also developed the RPA-CRISPR/Cas12a-LFDA to improve the test by making it more accessible for use and easier to read. The preliminary result showed that the RPA-CRISPR/Cas12a-LFDA could reliably distinguish between known positive and negative *Leptospira* spp. from clinical samples in our pilot study.

There were a number of strengths highlighted by this study, first we compared the test sensitivity, specificity, and accuracy at the day of fever, and the results showed that the time window after the onset of infection is a vital factor in the detection of positive infections in the different types of assays. Our newly developed test is capable of rapid and early detection of pathogenic *Leptospira* spp., which is critical for proper treatment. Second, our study was performed using blinded assessments and with three different observers to minimize bias. Third, we developed an LFDA to further facilitate field usage, and this pilot study achieved a similar LOD as the FBDA. Fourth, we performed a bioinformatics analysis to investigate nucleotide variation that would affect test sensitivity [7,37,38]. The results showed minimum variation in the *lipL32* targeted by crRNA of CRISPR/Cas12a among pathogenic *Leptospira* spp. As a result, there is still room for future improvement of our crRNA to ensure that it covers all pathogenic *Leptospira* spp. Finally, the RPA-CRISPR/Cas12a-based detection system is ideal for rural hospitals, as it has less expensive laboratory equipment requirements. The system would require only a heat block for the isothermal reaction. Additionally, the total time required to perform the RPA-CRISPR/Cas12a FBDA test, beginning with blood collection and DNA extraction, was approximately 1 hour and 35 minutes. Due to the extended reaction time required by CRISPR/Cas12a to completely cleave the probe, results from the RPA-CRISPR/Cas12a LFDA took approximately 2 hours compared to qPCR which takes 3–4 hours.

There were some limitations to our study, which must be acknowledged. The first consideration is that, due to the study design and variable timing of hospital admission, we cannot test all patients from the first day of fever. Therefore, the interpretation for the role of RPA-CRISPR/Cas12a in diagnosis leptospirosis need to understand this limitation. Second, most patients visited the hospital early after fever onset (first 3 day) and only a small number of patients presented at day 4–6 (n = 19) and more than 7 d (n = 18) after the fever onset. This time of presentation could impact test accuracy. As a result, we should interpret the results cautiously especially if the patients presented to the hospital after 7 days of fever onset. Third, the use of more than one target gene may enhance the efficiency of the test. While *lipL32* may contain a highly conserved domain, it is a single-copy gene, which may limit its sensitivity and LOD [39]. As a consequence, our future efforts should be directed toward developing additional target genes, particularly those with multiple copies, in order to increase the test’s LOD and sensitivity. Fourth, we conducted only a few cross-reactivity tests (n = 8). As a result, in our next investigation, we will undertake testing for known blood-borne diseases to confirm there is no cross-reactivity as well as testing against a broad variety of different *Leptospira* species. Fifth, we still do not know the stability/longevity/shelf life of the LFDA from Kestrel bioscience, Thailand. As a result, we will collaborate with Kestrel bioscience, Thailand, in the future to research the stability/longevity/shelf life of LFDA. Sixth, the qPCR CT greater than 37 had an effect on the test’s accuracy. Finally, clinical samples were collected from a single province in Thailand, which limits the generalizability of the findings.

Our study provides support for the use of the RPA-CRISPR/Cas12a-FBDA to detect leptospires due to its satisfactory LOD, sensitivity, and specificity. It is suitable for use in the field, especially in rural hospitals with limited resources since it is practical, portable, rapid, and simple to use. Only a heat box is required to perform the isothermal reaction. With the addition of the LFDA, we will be able to further decrease the need for equipment making the procedure even more desirable.

## Supporting information

S1 Table Inter-observer comparison. (DOCX)

S2 Table Bioinformatics analysis of the crRNA target sequence. (DOCX)

## References

[1] Costa F, Hagan JE, Calcagno J, Kane M, Torgerson P, Martinez-Silveira MS, et al. Global Morbidity and Mortality of Leptospirosis: A Systematic Review. PLoS Neglected Tropical Diseases. 2015;9(9):e0003898. doi: 10.1371/journal.pntd.0003898 26379143 PMC4574773

[2] Levett PN. Leptospirosis. Clinical Microbiology Reviews. 2001;14(2):296–326. doi: 10.1128/CMR.14.2.296-326.2001 11292640 PMC88975

[3] Soo ZMP, Khan NA, Siddiqui R. Leptospirosis: Increasing importance in developing countries. Acta Tropica. 2019;201:105183. doi: 10.1016/j.actatropica.2019.105183 31542372

[4] World Health O. Human leptospirosis: guidance for diagnosis, surveillance and control. Geneva: World Health Organization; 2003.

[5] Budihal SV, Perwez K. Leptospirosis Diagnosis: Competancy of Various Laboratory Tests. Journal of Clinical and Diagnostic Research 2014;8(1):199–202. doi: 10.7860/JCDR/2014/6593.3950 24596774 PMC3939550

[6] Ahmad SN, Shah S, Ahmad FM. Laboratory diagnosis of leptospirosis. Journal of Postgraduate Medicine. 2005;51(3):195–200. 16333192

[7] Narkkul U, Thaipadungpanit J, Srisawat N, Rudge JW, Thongdee M, Pawarana R, et al. Human, animal, water source interactions and leptospirosis in Thailand. Scientific Reports. 2021;11(1):3215. doi: 10.1038/s41598-021-82290-5 33547388 PMC7864926

[8] Ahmed A, van der Linden H, Hartskeerl RA. Development of a recombinase polymerase amplification assay for the detection of pathogenic Leptospira. International Journal of Environmental Research and Public Health. 2014;11(5):4953–64. doi: 10.3390/ijerph110504953 24814943 PMC4053868

[9] Dinhuzen J, Limothai U, Tachaboon S, Krairojananan P, Laosatiankit B, Boonprasong S, et al. A prospective study to evaluate the accuracy of rapid diagnostic tests for diagnosis of human leptospirosis: Result from THAI-LEPTO AKI study. PLoS Neglected Tropical Diseases. 2021;15(2):e0009159. doi: 10.1371/journal.pntd.0009159 33606698 PMC7894855

[10] Sonthayanon P, Chierakul W, Wuthiekanun V, Thaipadungpanit J, Kalambaheti T, Boonsilp S, et al. Accuracy of loop-mediated isothermal amplification for diagnosis of human leptospirosis in Thailand. American Journal of Tropical Medicine and Hygiene. 2011;84(4):614–20. doi: 10.4269/ajtmh.2011.10-0473 21460019 PMC3062458

[11] Daher RK, Stewart G, Boissinot M, Bergeron MG. Recombinase Polymerase Amplification for Diagnostic Applications. Clinical Chemistry. 2016;62(7):947–58. doi: 10.1373/clinchem.2015.245829 27160000 PMC7108464

[12] Gootenberg JS, Abudayyeh OO, Lee JW, Essletzbichler P, Dy AJ, Joung J, et al. Nucleic acid detection with CRISPR-Cas13a/C2c2. Science. 2017;356(6336):438–42. doi: 10.1126/science.aam9321 28408723 PMC5526198

[13] Gootenberg JS, Abudayyeh OO, Kellner MJ, Joung J, Collins JJ, Zhang F. Multiplexed and portable nucleic acid detection platform with Cas13, Cas12a, and Csm6. Science. 2018. 10.1126/science.aaq0179 PMC5961727 29449508

[14] Chen JS, Ma E, Harrington LB, Da Costa M, Tian X, Palefsky JM, et al. CRISPR-Cas12a target binding unleashes indiscriminate single-stranded DNase activity. Science. 2018. doi: 10.1126/science.aar6245 29449511 PMC6628903

[15] Daher Ede F, de Abreu KL, da Silva Junior GB. Leptospirosis-associated acute kidney injury. J Bras Nefrol. 2010;32(4):400–7. 21541455

[16] Fernandes LG, Siqueira GH, Teixeira AR, Silva LP, Figueredo JM, Cosate MR, et al. Leptospira spp.: Novel insights into host-pathogen interactions. Veterinary Immunology and Immunopathology. 2016;176:50–7. doi: 10.1016/j.vetimm.2015.12.004 26727033

[17] Haake DA, Chao G, Zuerner RL, Barnett JK, Barnett D, Mazel M, et al. The leptospiral major outer membrane protein LipL32 is a lipoprotein expressed during mammalian infection. Infection and Immunity. 2000;68(4):2276–85. doi: 10.1128/IAI.68.4.2276-2285.2000 10722630 PMC97414

[18] Evangelista K, Franco R, Schwab A, Coburn J. Leptospira interrogans Binds to Cadherins. PLoS Neglected Tropical Diseases. 2014;8(1):e2672. doi: 10.1371/journal.pntd.0002672 24498454 PMC3907533

[19] Schreier S, Triampo W, Doungchawee G, Triampo D, Chadsuthi S. Leptospirosis research: fast, easy and reliable enumeration of mobile leptospires. Biological Research. 2009;42(1):5–12. doi: /S0716-97602009000100001 19621128

[20] Sukmark T, Lumlertgul N, Peerapornratana S, Khositrangsikun K, Tungsanga K, Sitprija V, et al. Thai-Lepto-on-admission probability (THAI-LEPTO) score as an early tool for initial diagnosis of leptospirosis: Result from Thai-Lepto AKI study group. PLoS Neglected Tropical Diseases. 2018;12(3):e0006319. doi: 10.1371/journal.pntd.0006319 29554124 PMC5875898

[21] Villumsen S, Pedersen R, Borre MB, Ahrens P, Jensen JS, Krogfelt KA. Novel TaqMan(R) PCR for detection of Leptospira species in urine and blood: pit-falls of in silico validation. Journal of Microbiological Methods. 2012;91(1):184–90. doi: 10.1016/j.mimet.2012.06.009 22750039

[22] Srisawat N, Praditpornsilpa K, Patarakul K, Techapornrung M, Daraswang T, Sukmark T, et al. Neutrophil Gelatinase Associated Lipocalin (NGAL) in Leptospirosis Acute Kidney Injury: A Multicenter Study in Thailand. PloS One. 2015;10(12):e0143367–e. doi: 10.1371/journal.pone.0143367 26629810 PMC4667882

[23] Broughton JP, Deng X, Yu G, Fasching CL, Servellita V, Singh J, et al. CRISPR–Cas12-based detection of SARS-CoV-2. Nature Biotechnology. 2020;38(7):870–4. doi: 10.1038/s41587-020-0513-4 32300245 PMC9107629

[24] Mayuramart O, Nimsamer P, Rattanaburi S, Chantaravisoot N, Khongnomnan K, Chansaenroj J, et al. Detection of severe acute respiratory syndrome coronavirus 2 and influenza viruses based on CRISPR-Cas12a. Experimental Biology and Medicine. 2021;246(4):400–5. doi: 10.1177/1535370220963793 33153299 PMC7885046

[25] Smythe LD, Smith IL, Smith GA, Dohnt MF, Symonds ML, Barnett LJ, et al. A quantitative PCR (TaqMan) assay for pathogenic Leptospira spp. BMC Infectious Diseases. 2002;2(1):13. doi: 10.1186/1471-2334-2-13 12100734 PMC117785

[26] Nimsamer P, Mayuramart O, Rattanaburi S, Chantaravisoot N, Saengchoowong S, Puenpa J, et al. Comparative performance of CRISPR-Cas12a assays for SARS-CoV-2 detection tested with RNA extracted from clinical specimens. Journal of Virological Methods. 2021;290:114092. doi: 10.1016/j.jviromet.2021.114092 33539846 PMC7849546

[27] Caliendo AM, Hodinka RL. A CRISPR Way to Diagnose Infectious Diseases. New England Journal of Medicine. 2017;377(17):1685–7. doi: 10.1056/NEJMcibr1704902 29069564

[28] Li Y, Li S, Wang J, Liu G. CRISPR/Cas Systems towards Next-Generation Biosensing. Trends in Biotechnology. 2019;37(7):730–43. doi: 10.1016/j.tibtech.2018.12.005 30654914

[29] Kellner MJ, Koob JG, Gootenberg JS, Abudayyeh OO, Zhang F. SHERLOCK: nucleic acid detection with CRISPR nucleases. Nature Protocols. 2019;14(10):2986–3012. doi: 10.1038/s41596-019-0210-2 31548639 PMC6956564

[30] Abd El Wahed A, Patel P, Faye O, Thaloengsok S, Heidenreich D, Matangkasombut P, et al. Recombinase Polymerase Amplification Assay for Rapid Diagnostics of Dengue Infection. PLOS ONE. 2015;10(6):e0129682. doi: 10.1371/journal.pone.0129682 26075598 PMC4468249

[31] Picardeau M, Bulach DM, Bouchier C, Zuerner RL, Zidane N, Wilson PJ, et al. Genome sequence of the saprophyte Leptospira biflexa provides insights into the evolution of Leptospira and the pathogenesis of leptospirosis. PloS One. 2008;3(2):e1607. doi: 10.1371/journal.pone.0001607 18270594 PMC2229662

[32] Galloway RL, Hoffmaster AR. Optimization of LipL32 PCR assay for increased sensitivity in diagnosing leptospirosis. Diagnostic Microbiology and Infectious Disease. 2015;82(3):199–200. doi: 10.1016/j.diagmicrobio.2015.03.024 25912810 PMC6452440

[33] Kersting S, Rausch V, Bier FF, von Nickisch-Rosenegk M. Rapid detection of Plasmodium falciparum with isothermal recombinase polymerase amplification and lateral flow analysis. Malaria Journal. 2014;13:99. doi: 10.1186/1475-2875-13-99 24629133 PMC4004163

[34] Stoddard RA, Gee JE, Wilkins PP, McCaustland K, Hoffmaster AR. Detection of pathogenic Leptospira spp. through TaqMan polymerase chain reaction targeting the LipL32 gene. Diagn Microbiol Infect Dis. 2009;64(3):247–55. doi: 10.1016/j.diagmicrobio.2009.03.014 19395218

[35] Tubiana S, Mikulski M, Becam J, Lacassin F, Lefèvre P, Gourinat AC, et al. Risk factors and predictors of severe leptospirosis in New Caledonia. PLoS Negl Trop Dis. 2013;7(1):e1991. doi: 10.1371/journal.pntd.0001991 23326614 PMC3542117

[36] Picardeau M. Diagnosis and epidemiology of leptospirosis. Médecine et Maladies Infectieuses. 2013;43(1):1–9. doi: 10.1016/j.medmal.2012.11.005 23337900

[37] Murugan K, Seetharam AS, Severin AJ, Sashital DG. CRISPR-Cas12a has widespread off-target and dsDNA-nicking effects. J Biol Chem. 2020;295(17):5538–53. doi: 10.1074/jbc.RA120.012933 32161115 PMC7186167

[38] Lelu M, Muñoz-Zanzi C, Higgins B, Galloway R. Seroepidemiology of leptospirosis in dogs from rural and slum communities of Los Rios Region, Chile. BMC veterinary research. 2015;11:31-. doi: 10.1186/s12917-015-0322-z 25880871 PMC4329218

[39] Thaipadunpanit J, Chierakul W, Wuthiekanun V, Limmathurotsakul D, Amornchai P, Boonslip S, et al. Diagnostic Accuracy of Real-Time PCR Assays Targeting 16S rRNA and lipl32 Genes for Human Leptospirosis in Thailand: A Case-Control Study. PloS One. 2011;6(1):e16236. doi: 10.1371/journal.pone.0016236 21283633 PMC3026019

