# Supplementary material for: Rapid and sensitive point-of-care detection of Leptospira by RPA-CRISPR/Cas12a targeting lipL32
Source: PLoS Negl Trop Dis. 2022 Jan 6;16(1):e0010112. doi: 10.1371/journal.pntd.0010112 (PMC8769300; doi:10.1371/journal.pntd.0010112)
Supplement: S1 Table — (DOCX) [file pntd.0010112.s001.docx]

**S1 Table**. Inter-observer comparison.

| Parameter | Observer 1 | Observer 2 | Observer 3 |
| --- | --- | --- | --- |
| Sensitivity (%) | 87.04 | 87.04 | 85.19 |
| Specificity (%) | 100 | 100 | 100 |
| Accuracy (%) | 93.64 | 93.64 | 92.73 |
