# Supplementary material for: Rapid and sensitive point-of-care detection of Leptospira by RPA-CRISPR/Cas12a targeting lipL32
Source: PLoS Negl Trop Dis. 2022 Jan 6;16(1):e0010112. doi: 10.1371/journal.pntd.0010112 (PMC8769300; doi:10.1371/journal.pntd.0010112)
Supplement: S2 Table — (DOCX) [file pntd.0010112.s002.docx]

| ***Leptospira* spp.** | **Serovar/strain** | **crRNA target sequence** |
| --- | --- | --- |
| *L. interrogans* | Lai | TTTGTTCTGAGCGAGGACACAATC |
| *L. interrogans* | Australis | TTTGTTCTGAGCGAGGACACAATC |
| *L. interrogans* | Autumnali | TTTGTTCTGAGCGAGGACACAATC |
| *L. interrogans* | Copenhageni | TTTGTTCTGAGCGAGGACACAATC |
| *L. interrogans* | Canicola | TTTGTTCTGAGCGAGGACACAATC |
| *L. interrogans* | Hardjo | TTTGTTCTGAGCGAGGACACAATC |
| *L. interrogans* | Bataviae | TTTGTTCTGAGCGAGGACACAATC |
| *L. interrogans* | Icterohaemorrhagiae | TTTGTTCTGAGCGAGGACACAATC |
| *L. interrogans* | Pyrogenes | TTTGTTCTGAGCGAGGACACAATC |
| *L. interrogans* | Pomona | TTTGTTCTGAGCGAGGACACAATC |
| *L. interrogans* | Manilae | TTTGTTCTGAGCGAGGACACAATC |
| *L. borgpetersenil* | VG79 | TTTGTTCTGAGCGAGGACACAATC |
| *L. Kirschneri* | Tsaratsovo | TTTGTTCTGAGCGAGGACACAATC |
| *L. noguchii* | Pomona | TTTGTTCTGAGCGAGGACACAATC |
| *L. santarosai* | Shermani | TTTGTTCTGAGCGAGAGCACAGTT |
| *L. weilii* | Manhao II | TTTGTTCTGAGCGAGAGCACAGTT |

**S2 Table**. Bioinformatics analysis of the crRNA target sequence
